# Navigating the complexities of multimorbidity in primary health care

**DOI:** 10.1080/02813432.2025.2581949

**Published:** 2025-11-05

**Authors:** Anna Nager

**Affiliations:** Division of Family Medicine and Primary Care, Karolinska Institutet Sweden - NVS

Managing patients with multiple chronic conditions is a central responsibility for general practitioners (GPs). In daily practice, multimorbidity is more the rule than the exception, and the complexity it brings is familiar to most clinicians in primary care. While disease-specific guidelines are available, guidance for combinations of conditions is rare [1]. In these situations, clinical judgement and a pragmatic approach become essential.

The article collection, ‘Aspects and Challenges of Multimorbidity in Primary Health Care’, presents studies that explore different facets of multimorbidity. The contributions include surveys of general practitioners, registry-based analyses, patient questionnaires, and qualitative interviews. Together, they offer insights into multimorbidity and how it is managed in primary care in the Nordic countries, and highlight areas where further development may be needed.

## Vulnerable groups and everyday practice

Two patient groups stand out in the studies: the oldest old, and those living with both chronic physical conditions and mental illness. These groups often require particular attention and adaptation of care.

The article ‘Hypertension management in the oldest-old: a survey of physicians in Swedish primary health care’ demonstrates that many general practitioners in Sweden do, in fact, find national and regional guidelines useful for the management of hypertension among the oldest old, even though clearer guidelines for treating multimorbid patients were requested from a high proportion of GPs. The study also shows that approximately half of GPs frequently discontinue antihypertensive treatment due to patients’ cognitive or physical deterioration. When asked to choose which is more important in the consideration of hypertension treatment—medical factors or living conditions—most GPs selected living conditions [2].

A register-based study from Finland found that frequent attenders (>7 face-to-face visits to a doctor or nurse in primary health care per year) accounted for 16% of all patients, but nearly half of all the visits. However, only 0.6% were frequent attenders over a five-year period. Frequent attendance was closely linked to chronic disease and multimorbidity, which increased with attendance frequency. Among individual disease groups, chronic skin wounds showed the strongest association with frequent attendance. Other disease categories exhibiting the most pronounced associations with frequent attendance among 5-year frequent attenders were psychotic and bipolar disorders, personality disorders, migraine and other headache syndromes, and obesity.

## Mental health and chronic disease

Mental illness is a certain risk for several chronic disorders. For instance, individuals with COPD who also experience depression and/or anxiety are at heightened risk of exacerbations, and increased morbidity and mortality [3–5]. In an article in the collection based on a patient survey among patients with COPD in Sweden, 29% women and 16% men reported depression/anxiety. Depression and anxiety were associated with younger age, current smoking, comorbid asthma, and dyspnea. The authors speculate that the higher prevalence of depression/anxiety in younger patients with COPD may be related to higher demands on functionality and health in general in younger people and possibly underreporting of depression/anxiety among older patients. Probably, there is a bidirectional pathway between depression/anxiety and COPD outcomes, involving physical activity and adherence to treatment [6].

One reason for poorer outcomes in patients with chronic conditions and depressive symptoms may be difficulty with self-management. A patient survey conducted in northern Sweden among individuals in their seventies with long-term health conditions demonstrated a strong association between experiencing depressive symptoms or poor health status and difficulties with self-management. These findings suggest that person-centred and holistic care, alongside continuity of care, are particularly important in this group to support effective self-management [7].

Another study in the article collection explored problem-solving therapy in general practice for patients with poor mental well-being and the chronic diseases type 2 diabetes and ischemic heart disease. The study, conducted across four general practices in Denmark, involved ethnographic observation as well as qualitative interviews with both patients and healthcare professionals. The results indicate that when healthcare professionals adapted services to specific patient needs, patients felt welcomed and accepted. Furthermore, finding a balance between letting the patient take responsibility while still providing sufficient support led to patients feeling empowered while still feeling cared for [8].

## Quality of life and multimorbidity

The quality of life is an aspect of multimorbidity highlighted in the article collection. Unsurprisingly, there is an association between higher levels of multimorbidity and reduced quality of life. A register-based study from northern Finland followed individuals born in 1966 between ages 31 to 46. The study found that, overall, health-related quality of life declined over the 15-year follow-up period for all participants. Moreover, the reduction in quality of life was significantly associated with multimorbidity [9].

Needs-based quality of life is a broader concept than health-related quality of life, encompassing all aspects of life satisfaction based on personal needs. In a large patient survey on patients in Danish primary care, a cumulative burden of multimorbidity was identified as an increasing number of diagnoses was associated with lower needs-based quality of life [10].

## Towards improved multimorbidity care

The studies in this collection show the importance of treating the whole person, not just individual diseases. Living conditions and social context are integral to good multimorbidity care. Overlooking mental illness in patients with chronic conditions may lead to poorer outcomes, whereas supporting self-management can help by enabling patients to take an active role in their health. Primary care needs to be organised so that multimorbidity is properly addressed with these aspects in mind.

## Use of AI

The AI tools ChatGPT and Google Gemini were used to enhance grammar and language.
